# Development and preliminary evaluation of a rehabilitation consult for survivors of head and neck cancer: an intervention mapping protocol

**DOI:** 10.1186/s13012-014-0191-z

**Published:** 2015-01-09

**Authors:** Sara E McEwen, Aileen M Davis, Jennifer M Jones, Rosemary Martino, Ian Poon, Ana Maria Rodriguez, Jolie Ringash

**Affiliations:** St. John’s Rehab Research Program, Sunnybrook Research Institute, 285 Cummer Avenue, Toronto, ON M2M 2G1 Canada; Department of Physical Therapy and Graduate Department of Rehabilitation Science, University of Toronto, 160-500 University Avenue, Toronto, ON M5G 1V7 Canada; Toronto Western Research Institute, University Health Network, 399 Bathurst Street, Toronto, ON M5T 2S8 Canada; Institute of Health Policy, Management and Evaluation and Institute of Medical Science, University of Toronto, Toronto, ON Canada; Cancer Survivorship Program, Princess Margaret Cancer Centre, University Health Network, 200 Elizabeth Street, Toronto, ON M5G 2C4 Canada; Department of Psychiatry, University of Toronto, Toronto, ON Canada; Department of Speech Language Pathology, University of Toronto, 160-500 University Avenue, Toronto, ON M5G 1V7 Canada; Department of Radiation Oncology, Odette Cancer Centre, Sunnybrook Health Sciences Centre, 2075 Bayview Avenue, Toronto, ON M4N 3M5 Canada; Department of Radiation Oncology, University of Toronto, Toronto, ON Canada; School of Physical and Occupational Therapy, McGill University, 3654 Promenade Sir William Osler, Montreal, QC H3G 1Y5 Canada; Department of Radiation Oncology, Princess Margaret Cancer Centre, University Health Network, 610 University Avenue, Toronto, ON M5G 2M9 Canada

**Keywords:** Head and neck cancer, Rehabilitation, Intervention mapping, Protocol, Self-management, Goal setting

## Abstract

**Background:**

Evidence suggests that rehabilitation interventions can improve function and quality of life in survivors of head and neck cancer (HNC), but there is a lack of coordinated, integrated services, and those offered are inconsistent. To address these gaps, we will develop and conduct preliminary evaluation of a rehabilitation consult, built on the theoretical foundations of goal setting and self-management, and composed of a brief functional evaluation, a resource compendium, and collaborative goal-setting and action planning processes.

**Methods/design:**

The development of the rehabilitation consult will be guided by intervention mapping, which consists of six steps: 1. Needs assessment; 2. Definition of program objectives; 3. Selection of theory-based intervention methods; 4. Production and pretesting; 5. Adoption, implementation and sustainability planning; 6. Process and effect evaluation. Within the intervention mapping framework, an iterative process of constructing drafts and mini-evaluations with consumers and experts will be used, modifying the rehabilitation consult intervention until a version suitable for formal evaluation is established. The rehabilitation consult will then be evaluated using a prospective, mixed method, single group design with 30 survivors of head and neck cancer. Outcomes will be assessed pre- and post-intervention and at 6-month follow-up.

**Discussion:**

Survivors of head and neck cancer have among the most complex rehabilitation needs of all cancer patients. The rehabilitation consult is expected to improve knowledge and uptake of rehabilitation resources and strategies in survivors of head and neck cancer and thereby improve long-term function and quality of life. If the rehabilitation consult is effective in cancer patients with such high and diverse needs, this project will produce a toolkit that will be adaptable for other types of cancer in other jurisdictions.

## Background

In contrast with other major chronic conditions such as heart disease and stroke, cancer care does not routinely integrate evidence-based rehabilitation services within the standard continuum. This protocol describes a structured process for the development, implementation and preliminary evaluation of a novel, integrated rehabilitation intervention for survivors of head and neck cancer (HNC) called the rehabilitation consultation (RC). The RC program goals are to increase knowledge about rehabilitation needs and resources to meet those needs, to establish individualized rehabilitation goals for HNC survivors and personalized action plans to meet those goals and to provide support to HNC survivors for the implementation and evaluation of action plans. The goals will be personally important to the individual survivor, and the action plans will be achievable, using resources they can access close to home rather than at the cancer centre, when possible. Additionally, the RC will be integrated into routine HNC follow-up procedures and will be administered as soon as possible following active cancer treatment. It will be led by a professional with a background in one of the traditional rehabilitation professions and will include the following three components: 1. A brief, HNC-specific functional evaluation; 2. A resource compendium; 3. Collaborative goal-setting, action-planning and follow-up processes.

Reductions in function and quality of life are particularly high in HNC, as the disease and treatment cause more diverse and serious impairments than many other cancers. Issues may include reductions in swallowing, speech, neck and upper extremity mobility, general deconditioning, fatigue, insomnia, lymphedema, neuropathies, visible facial deformity and psychological distress [1-9]. In addition, a range of more global functional issues result, including body image dissatisfaction, cognitive and behavioural problems [10], decreased role functioning [11], decreased nutritional status [12], decreased communication [5], poor driving performance [13] and inability to return to work [14,15]. Among the 9,000 new cases of HNC in Canada each year [16], an increasing proportion is among young, working-aged patients, primarily related to the ongoing epidemic of oropharyngeal cancer associated with Human Papillomavirus [17]. Without rehabilitation services, the influx of younger survivors may increase the societal burden of the illness through loss of employment, increased absenteeism and the financial, social and potentially emotional effects on their families which may include young dependents [18,19].

Cancer rehabilitation has been defined as coordinated, professional care designed to enable people to maximize physical, social and psychological function within the limits imposed by the disease and treatment effects and to engage in personally valued activities within their social contexts [20]. Rehabilitation interventions for HNC are demonstrably safe, feasible, cost-effective and associated with improvements in quality of life, general conditioning, swallowing, muscle function, insomnia, pain, weakness, anorexia, shortness of breath, tube-feeding dependency, hospital readmissions, depression and distress [7,21-25]. Although evidence exists to support cancer rehabilitation, services are fragmented. Rehabilitation professionals are consulted infrequently and often long after treatment ends, when chronicity of problems limits the impact of intervention [unpublished observations]. Additional barriers to accessing rehabilitation services include cost, issues between and among patients, oncology professionals and rehabilitation experts related to communication and awareness of available resources [unpublished observations]. There are clear potential benefits to a comprehensive, integrated rehabilitation consultation process that targets all HNC patients soon after primary cancer treatment is completed, improves communication among stakeholders and provides linkages to appropriate resources. Therefore, the objective of this project is to develop, implement and conduct a pilot evaluation of the RC.

## Methods/design

This project employs intervention mapping as an ecologically valid, structured framework to develop, implement and evaluate the RC [26]. Intervention mapping consists of six steps: 1. Needs assessment; 2. Definition of program objectives; 3. Selection of theory-based intervention methods; 4. Production and pretesting; 5. Adoption, implementation and sustainability planning; 6. Process and effect evaluation. Research staff and the investigators will oversee the project with input from an eight-member advisory panel, including patient and family representatives, health care professionals working in oncology, health care professionals working in rehabilitation and representatives of the provincial cancer care system. All intervention mapping steps are described below. Note that steps 1 and 2 have previously been completed, with steps 3–6 planned or in progress.

In the previously completed step 1, *needs assessment*, we used information gleaned from focus groups with patients, family members and front-line health care professionals (unpublished data, manuscript under preparation) and from a scoping literature review (unpublished data, manuscripts under preparation) to establish the rehabilitation needs of HNC survivors. We conducted six focus groups with 38 survivors, family members and health care professionals from two large cancer centres. Using directed content analysis of the transcripts, we answered specific research questions, including “What are the functional issues following treatment for HNC that might be mitigated with rehabilitation interventions?” Functional issues identified were categorized as impairments or function/participation/health issues and formed the basic list of rehabilitation needs. To augment that list, a scoping literature review was conducted to answer the question, “What is known from the existing literature about outcomes of HNC survivors that are potentially amenable by rehabilitation interventions?” Relevant observational studies published between 2003 and 2013 were identified from MEDLINE, EMBASE, CINAHL, PsycINFO and RehabDATA databases. An initial 1,245 abstracts were screened, 422 full text manuscripts were read and 74 observational studies were identified for inclusion in the review. Observed impairment (body functions and body structures), function, participation and health outcomes deemed as potentially amenable to rehabilitation interventions were added to the list of rehabilitation needs developed from the focus groups. The rehabilitation needs appear as phase 2 and phase 1 in Table 1. We next identified highly relevant and modifiable behavioural and environmental factors that contributed to the identified issues (Phase 3, Table 1), their determinants (Phase 4, Table 1) and the items targeted for change with the RC intervention (items in italics in Table 1). Finally, the following RC program goals were developed: increase knowledge of all stakeholders about rehabilitation needs and about resources to meet those needs, establish individualized rehabilitation goals and personalized action plans for HNC survivors, provide support for the implementation and evaluation of action plans and facilitate HNC survivors’ access to rehabilitation professionals where it is most feasible for them.

**Table 1:** **Intervention mapping step 1, needs assessment logic model**

	**Phase 4: determinants**	**Phase 3: behavioural and environmental factors**	**Phase 2: body structures and functions**	**Phase 1: function, participation, and health**
Modifiable	*Knowledge about condition*	*Goal setting and action planning*	Swallowing	*Decreased functional status,*
			Fatigue	
	*Knowledge about symptom management and resources*	*Health care professionals knowledge about rehab and HNC treatment*	Muscle strength, joint mobility	*Decreased health status, Decreased participation and engagement in personally-meaningful life areas*
			Cardiovascular capacity	
	*Knowledge about metacognitive strategy use*	*Expectations*	Dental issues	
		*Energy budgeting*	Mouth opening	
			Neck and arm range of motion	*Decreased psychosocial function*
	Knowledge about association among needs/issues?	Coping habits	Speech	
		*Exercise habits*	Anxiety	*Decreased role function*
		*Dietary habits*	Dry mouth	
		*Substance use, particularly smoking*	Neuropathies/sensory issues	*Decreased employment*
	Local processes		Fibrosis	
	Physician referral processes	*Action planning*	Pain	
		*Problem solving*	Cardiovascular capacity	
		*Developing strategies*		
	*Self efficacy*			
	Communication style	*Self regulation/self evaluation*	Digestive system functioning	
	Coping style	*Rehab resources*	Weight loss	
	Pre-existing mood/behaviour	Community resources	Cognitive impairments	
		*Access to resources*	Personality changes	
			Emotional issues	
			Intimacy	
			Body image/body satisfaction	
			Lymphedema	
			Late effects	
Non-modifiable	Disease site	Hospital type (eg cancer centre vs tertiary care centre)	Aesthetic outcomes	
	Treatment received			
	Age			
	Sex			
	Education level			
	Socioeconomic status/finances			
	Personality			

Items in *italics* are targeted for change.

In the previously completed step 2, *definition of program objectives*, the resource requirements that will enable achievement of the program goals described above were specified (see Table 2). The specific resource requirements to be developed as components of the RC include a brief, HNC-specific functional evaluation, an online resource compendium that includes comprehensive information about rehabilitation services in Toronto and adjacent regions as well as educational modules for specific home-based exercises, a goal-setting and action planning process and a follow-up process. The specific behavioural requirements are that the HNC survivors be confident in goal-setting and action planning and that all stakeholders be knowledgeable about HNC rehabilitation needs and applicable resources.

**Table 2:** **Intervention mapping step 2, change objectives logic model**

**Change objective**	**Determinants**	**Performance objectives**	**Behavioural outcomes**	**Body functions and structure**	**Function, participation and health**
Resources will be available (rehab needs assessment and criteria, online resource compendium, goal setting process) Survivor’s will be confident in goal-setting and action plan development	Sufficient cognitive and communication skills	The rehab professional will assess HNC survivors for rehab needs	Of those survivors who participate in the RC, those with rehab concerns will set rehab goals and action plans.	Cognition, self-efficacy	Improved engagement
	Individual impairment				
	Level of anxiety/depression/motivation	Rehab professional will use criteria to determine those with rehab needs (triage?) Survivor will set goals and action plans, facilitated by rehab professional			
	Baseline level of self-efficacy				
Follow-up process will be in place	Disease site and progression	Survivors will follow through with the action plan. If there is an issue, the survivor will develop a new action. The rehabilitation professional will follow up to discuss the state of implementa-tion of the action plan, and will guide the survivor to the next steps as necessary. Subsequent follow-ups will be scheduled as necessary.	Those with rehab concerns will follow through on action plan and modify it when necessary (with support).	Cognition, self-efficacy, and reduction of targeted functional issues (depends on the individual)	Improved engagement, function, participation, and quality of life
	Age				
	Comorbidities				
	Cognitive skills				
	Communication skills				

**Figure 1:**
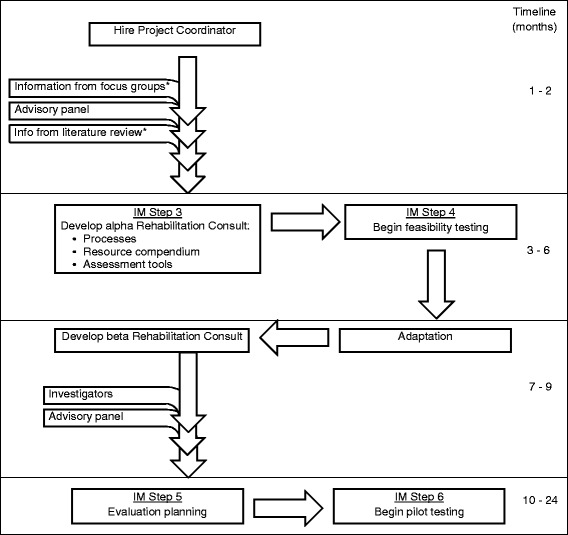
**Rehabilitation consult (RC) development flow chart and timeline.** *Literature review and focus group data were collected in a previous project; *IM* intervention mapping.

Figure 1 provides a visual overview of the current project, intervention mapping steps 3 through 6. In step 3, *selection of theory-based intervention methods and practical applications*, the research team will review the work completed in intervention mapping steps 1 and 2 with the advisory panel, with a particular emphasis on the RC program goals and ideas. Then, the research team will select behavioural change methods from the intervention mapping tables ([26], p 357–358) that are congruent with self-management and goal-setting theories and with the RC program parameters. Behavioural change methods will be identified for specific change objectives at both the level of the individual and at the level of the environment. The methods to be examined for change at the individual level will include, for example, methods to change skills, capabilities and self-efficacy and overcome barriers; for change at the environmental level, methods to change organizations is an example. From all potential methods identified, those that best match the RC parameters will be selected. The research team will then identify, using facilitated brainstorming, practical applications (such as worksheets, pamphlets, websites and videos) to enact those methods. The advisory panel will conduct a final review of all methods and practical applications selected to ensure that they mesh with the program goals and resource requirements identified in intervention mapping steps 1 and 2 and will make recommendations for modifications if necessary.

Self-management is the process of learning the skills necessary to independently lead an active and satisfying life when faced with a chronic condition [27]. It is associated with improved functional and quality of life outcomes in numerous conditions [28] and is recommended as a means to reduce health costs [29-31]. While traditional, didactic patient education programs demonstrate improvements in knowledge, they generally do not lead to changes in behaviour or to improvements in health [27,32]. A self-management approach, on the other hand, is associated with improved health behaviours [33], likely through the process of evoking executive cognitive processes such as goal setting, action planning and self-evaluation. Preliminary evidence suggests self-management interventions for people living with cancer are efficacious [34]. Self-management is particularly useful to promote complex behavioural change, such as modification of exercise, diet and substance use [26].

A collaborative, patient-centred approach to goal setting, in which a facilitator actively encourages the participation of the patient, has long been espoused in rehabilitation. There is evidence to suggest that goal achievement is improved when this approach is employed [35,36]. As well, goal setting is believed to influence adherence to rehabilitation programs [37]. The involvement of the patient ensures the goals are personally meaningful, thereby increasing his or her motivation to act. In the Rubicon model of action [38], it is proposed that while motivation and action are inextricably linked, they are separate entities. Initially, a wish or need is identified and considered but may not be acted upon. However, the act of forming a specific, concrete goal may provide the momentum needed to move to planning and then action; as the name of the model suggests, the Rubicon is crossed, and turning back is unlikely.

In step 4, *production of program components and pre-testing*, all components of the RC will be fully developed and pre-tested for acceptability and feasibility. To gather information for the resource compendium, we will conduct telephone interviews with key informants from Toronto and the adjoining regions that commonly refer HNC patients to Toronto. A snowball recruitment technique will be used to determine key informants, in which an initial list is established, and each of those is asked to recommend additional informants, and so on. The brief HNC-specific functional evaluation has not only the objectives of determining both performance-based issues, such as swallowing or joint mobility, but also patient-determined functional and life participation issues, such as return to work or family and social role functioning. The evaluation is intended to have a combination of observed physical assessment items and patient-reported indicators. Informed by the prior literature review, the investigators and study staff will generate an initial list of items that will be reviewed for content, length and intelligibility by the advisory panel and modified as necessary. Similarly, the investigators and study staff will initially develop all processes, such as how to efficiently integrate the evaluation into routine follow-up, which will then be reviewed by the advisory panel and modified as necessary.

Once an alpha version of the RC is developed, a convenience sample of 10 multidisciplinary clinicians and 10 post-treatment HNC survivors will be recruited from the Princess Margaret Cancer Centre (PMCC) and Odette Cancer Centre to assess its feasibility and acceptability. Based on the findings, further modifications will be made, resulting in a beta version of the RC that is ready for implementation and evaluation.

Step 5, *planning for adoption, implementation, and sustainability,* is a planning step, in which a logic model will be developed to guide the initial implementation and program evaluation. Specific objectives for adoption, implementation and sustainability will be established, and determinants of those will be considered.

Step 6, *evaluation*, will implement the program evaluation plan established in step 5 and will also include a pilot outcome evaluation to estimate the impact of the RC on function and quality of life. We will implement and evaluate the RC using a mixed method, single group study with a convenience sample of approximately 30 HNC survivors post primary cancer treatment, recruited from the PMCC HNC clinics. Eligible participants will be adult survivors of HNC who have completed active treatment (surgery, radiation, chemotherapy or any combination thereof) within the past 1–4 months. Exclusion criteria will be lack of English fluency or concurrent major degenerative conditions likely to cause functional deterioration. A research assistant not involved with clinical administration of the RC will assess the following constructs pre- and post-RC intervention and at a follow-up 6 months later: function, quality of life, self-efficacy, community participation, goal attainment and return to work status (if applicable). Table 3 describes all standardized tools to be used. To explore survivor experiences with the RC, one-on-one semi-structured interviews will be administered at the 6-month follow-up.

**Table 3:** **Outcome measures**

**Construct**	**Instrument(s)**	**Description and pyschometric properties**
Health-related quality of life	Medical Outcomes Study 36-item Short-Form Health Survey (SF-36) [39,40]	SF-36 a widely used, generic, patient-report measure created to assess health-related quality of life (HRQOL). It consists of eight domains: physical functioning, role limitations due to physical problems, bodily pain, general health perceptions, social functioning, general mental health, role limitations due to emotional problems, and vitality. SF-36 has been widely tested, and, with the exception of the social functioning subscale, has excellent internal consistency and interrater reliability; SF-36 has adequate to excellent convergent validity with a number of functional and HRQoL scales.
	The Functional Assessment of Cancer Therapy-Head and Neck Version 4 (FACT-H&N) [41]	Self-report reliable and valid quality of life questionnaire. The scale consists of a core FACT-G (General) questionnaire that covers four domains: physical, social/family, emotional, and functional. The scale is supplemented by a head and neck cancer specific subscale. Items are rated on a 0 (*Not at all*) to 4 (*Very much*) Likert scale and scores are calculated to produce subscale scores for each domain. It is reliable and valid in patients with HNC, scores correlating with treatment status and global performance status.
Participation	Reintegration to Normal Living Index (RNL) [42]	The RNL consists of 11 items covering areas such as recreational and social participation, community mobility, family roles and other relationships. It has high internal consistency, moderate interrater reliability and is correlated with measures of quality of life and well being.
Self-efficacy	Self-Efficacy Gage (SEG) [43]	The SEG asks participants to rate 28 functional activities on a 10-point scale of how confident they are to complete the activity without the help of another person, with 1 indicating “not confident at all” and 10 indicating “completely confident”. The SEG has excellent internal consistency (*r* = 0.94), test-retest reliability (0.90), and convergent validity has been demonstrated through significant correlations with occupational performance.
Return to work	Radiation Therapy Oncology Group (RTOG) Work Status Questionnaire [44]	The RTOG Work Status Questionnaire is a brief, patient-report tool that takes less than 5 min to complete. It was designed for use in RTOG trials, and psychometric properties have not been tested but meet content validity criteria and sensibility criteria.
Goal attainment	Goal Attainment Scaling (GAS) [45,46]	The GAS is a measure that allows the comparison of individual progress towards personal goals between participants. Patients’ level of attainment are rated on a five –point scale from −2 (*much less than expected)* to +2 (*much more than expected*). Patients who obtain a score of zero or higher are considered to have achieved their goals. The GAS has been shown to have high interrater reliability, good content validity, good responsiveness, and to be responsive to clinically important change.

Quantitative data analysis will be exploratory and descriptive, and effect sizes will be calculated for all outcomes to help plan for a future, controlled trial. We will calculate means, standard deviations and Cohen’s d [47] effect size for normally distributed data. For non-normally distributed data, we will calculate medians, ranges and a nonparametric effect size *r* using the formula *r*^2^ = *z*^2^/*N* [48]. For qualitative analysis, all interviews will be audio recorded, transcribed verbatim and analysed using a two-phased, hybrid approach that is both deductive and inductive, described by Fereday and Muir-Cochrane [49]. In the first phase, directed, deductive extraction of data elements that answer pre-determined questions regarding survivor experiences with the RC components will occur. In the second phase, data will be re-analysed inductively to recognize patterns in the data not previously anticipated that may help to enhance the RC. Both quantitative and qualitative findings will be summarized and reported to the advisory panel, who will then make recommendations regarding any additional modifications to the RC. The research team will make final decisions about RC modifications and will finalize a version for future evaluation.

Assuming positive outcomes from this single group evaluation, a future, multi-site controlled trial will be designed and implemented. Results from this study will provide feasibility information, such as recruitment rates, will help to define primary and secondary outcomes and will provide data to calculate sample size to ensure an adequately powered trial.

## Discussion

This project brings together the diverging views of rehabilitation specialists, focused on long-term real-world function, with those of cancer specialists, focused on acute treatment and episodic symptom management. We have set out to bring those views together to develop a clinically effective and cost-effective rehabilitation intervention that integrates seamlessly with an existing cancer care system. Survivors of HNC have among the most complex rehabilitation needs of all cancer patients because of the anatomical complexity of the head and neck region. The RC is expected to improve knowledge and uptake of rehabilitation resources and strategies in survivors of HNC and thereby improve function and quality of life. The RC will be designed to ensure the components are readily modifiable for use beyond the regional cancer centres within which they were developed. Further, we believe that HNC serves as an ideal incubator for development of the RC. If it is effective in cancer patients with such high and diverse needs, it is expected that this project will produce a toolkit that will be adaptable for other types of cancer in other jurisdictions.

## Abbreviations

HNC: Head and neck cancer
PMCC: Princess Margaret Cancer Centre
RC: Rehabilitation consult
